# Multi-volume modeling of *Eucalyptus* trees using regression and artificial neural networks

**DOI:** 10.1371/journal.pone.0238703

**Published:** 2020-09-11

**Authors:** Gileno Brito de Azevedo, Heitor Vicensotto Tomiazzi, Glauce Taís de Oliveira Sousa Azevedo, Larissa Pereira Ribeiro Teodoro, Paulo Eduardo Teodoro, Marcos Talvani Pereira de Souza, Tays Silva Batista, Humberto de Jesus Eufrade-Junior, Saulo Philipe Sebastião Guerra

**Affiliations:** 1 Federal University of Mato Grosso do Sul (UFMS), Chapadão do Sul, MS, Brazil; 2 College of Agricultural Sciences (FCA), Sao Paulo State University (UNESP), Botucatu, SP, Brazil; Newcastle University, UNITED KINGDOM

## Abstract

The stem volume of commercial trees is an important variable that assists in decision making and economic analysis in forest management. Wood from forest plantations can be used for several purposes, which makes estimating multi-volumes for the same tree a necessary task. Defining its exploitation and use potential, such as the total and merchantable volumes (up to a minimum diameter of interest), with or without bark, is a possible work. The goal of this study was to use different strategies to model multi-volumes of the stem of eucalyptus trees. The data came from rigorous scaling of 460 felled trees stems from four eucalyptus clones in high forest and coppice regimes. The diameters were measured at different heights, with the volume of the sections obtained by the Smalian method. Data were randomly separated into fit and validation data. The single multi-volume model, volume-specific models, and the training of artificial neural networks (ANNs) were fitted. The evaluation criteria of the models were: coefficient of determination, root mean square error, mean absolute error, mean bias error, as well as graphical analysis of observed and estimated values and distribution of residuals. Additionally, the t-test (α = 0.05) was performed between the volume obtained in the rigorous scaling and estimated by each strategy with the validation data. Results showed that the strategies used to model different tree stem volumes are efficient. The actual and estimated volumes showed no differences. The multi-volume model had the most considerable advantage in volume estimation practicality, while the volume-specific models were more efficient in the accuracy of estimates. Given the conditions of this study, the ANNs are more suitable than the regression models in the estimation of multi-volumes of eucalyptus trees, revealing greater accuracy and practicality.

## Introduction

Currently, forest plantations in Brazil occupy about 9.85 million hectares of the planted area [1], being 6.73 certified by FSC and/or PEFC, with revenues that reach 1.1% of the gross domestic product (GDP), corresponding to U$ 18.5 billion [2]. The forest sector is increasingly consolidated, and along with its growth, the need to know the characteristics of forest plantations and their growth stocks increases. In Brazil, eucalyptus wood is used for several purposes, such as the production of firewood, coal, cellulose, wood panels, stakes, posts, wood for sawmills, among others. For each purpose, the wood must have specific dimensions. Thus, in plantations where wood is intended for multiple uses, using appropriate techniques to accurately estimate the sustainable production potential of wood for different purposes is paramount.

Stem volume is one of the essential measures to know the wood potential of the forest plantations, considering that this variable provides subsidies for the evaluation of the wood stock and the analysis of the sustainable yield potential of the forests [3–7]. In Brazil, the most commonly used procedure for obtaining tree volume in forest plantation is the volumetric equations. The Schumacher and Hall [8] model has been the primary model [6, 9, 10], besides the Taper equations in some cases [10, 11]. These equations allow the estimation of volume (total, or even a minimum diameter of interest), which is difficult to be directly obtained from variables that are more easily measured in the field, such as diameter at breast height and the total height of the trees [6, 12].

Despite the efficiency of volumetric equations in estimating wood volume [13, 14], they may have limited properties for some situations. For instance, when the goal is to estimate more than one volume for trees, such as total and merchantable volumes (up to a minimum diameter of interest), with or without bark, these volumetric equations are not efficient. With the ever-changing market conditions, the accurate estimate of tree volumes to different upper stem merchantability limits is paramount [4]. Nevertheless would require several equations, one for each volume of interest [15–17]. In turn, the Taper equations allow estimating the diameter at any stem height, the commercial height that occurs in a specific diameter, and the volume between stem sections. These estimates are adequate when the goal is to quantify multi-products of the tree [11, 18, 19]. A disadvantage of Taper equations, though, is the impossibility of estimating volumes with and without bark simultaneously. This information is desired, depending on the purpose of production and management.

In this sense, multi-volume models were developed to estimate different volumes, using a single equation [16, 20–22]. The multi-volume model developed by Leite et al. [20] was based on the expansion of the Schumacher and Hall [8] model and consisted of a volumetric model to simultaneously estimate the total and merchantable volumes (in different tree diameters), with and without bark. Adjusting these models can result in more available volume estimation keeping the accuracy, compared with traditional volumetric models, and maintaining compatibility properties between the total volume and the other parts [16, 20, 22, 23].

In general, estimators of volumetric and multi-volume models are obtained by regression techniques [6, 14, 17, 20, 24]. However, Artificial Neural Networks (ANNs) is an alternative that has been widespread in forest management to obtain tree volume estimates [5, 16, 25–33]. ANNs training provides linear and non-linear models based on mathematical estimates obtained identically to the logic of human reasoning [34].

Artificial intelligence tools have been increasingly adopted over the past 20 years to overcome problems related to the lack of conventional statistical assumptions, where foresters need to deal with noisy multidimensional data that are strongly non-linear [25, 35]. RNAs provide a specific approach for developing predictive models, offering a powerful method to analyze complex relationships between variables, without having to make assumptions about the data, which creates significant advantages over the use of conventional regression models [35]. Therefore several studies have shown advantages in employing ANNs in forestry measurement [26, 36–40].

Leite et al. [20] proposed a strategy for estimating multiple volumes of the stem that allows considering the presence or not of bark and different minimum commercial diameters predictor variables. However, to this day, the present study is the first one to address the efficiency of ANNs to predict multi-volumes of the stem. This work aims to use different strategies to model multi-volumes of the stem of eucalyptus trees. The hypotheses of this study were: (1) the multi-volume single model proposed by Leite et al. [20] provides similar estimates to volume-specifics models, proposed by Schumacher and Hall [8], separately adjusted for each volume of interest (total and a merchantable volume, with and without bark); (2) the ANNs are efficient to estimate multi-volumes of the stem.

## Material and methods

### Study area

Data were obtained from four eucalyptus clones (AEC0144—*Eucalyptus urophylla*, AEC0224—*Eucalyptus urophylla*, VM01—*Eucalyptus urophylla* × *Eucalyptus camaldulensis* and H77—*Eucalyptus urophylla* × *Eucalyptus grandis*), grown at an average planting spacing of 3 x 3 m, in the high forest and coppice scheme (first and second rotation). In Brazil, these clones are used for several purposes. The plantations are located in Ribas do Rio Pardo, State of Mato Grosso do Sul, Brazil, at an average elevation of 415 m, in the geographic coordinates 20º27'15" S and 53º19'60" W. The climate is classified, according to Köppen, as Aw tropical, with annual temperature and rainfall averages of 24.1°C and 1425 mm, respectively [41]. The silvicultural and dendrometric characteristics of the plantations are shown in Table 1.

**Table 1:** Average silvicultural and dendrometric characteristics of the evaluated plantings.

Clone	AEC0144	AEC0144	AEC0224	AEC0224	VM01	VM01	H77	H77
Rotation	1st	2nd	1st	2nd	1st	2nd	1st	2nd
Planting age (years)	6.8	13.6	6.6	9.5	6.3	12.7	14.5	13.5
Sprouting conduction age (years)	-	6.0	-	3.1	-	5.7	-	5.9
DBH (cm)*	17.8±2.9	14.6±3.5	18.8±3.0	13.7±2.6	16.7±2.9	13.4±5.3	20.6±5.3	16.4±4.8
Height (m)*	23.5±2.9	23.7±4.0	24.1±4.0	18.3±2.5	19.9±2.9	17.9±5.7	27.9±3.0	19.2±3.9
N. stems by hectare*	987±42	1703±93	933±30	1088±9	1049±59	1635±73	976±57	1067±212
N. stems by stump*	1.00±0.00	1.56±0.09	1.00±0.00	1.16±0.08	1.00±0.00	1.59±0.04	1.00±0.00	1.39±0.10
Basal area (m^2^ ha^-1^)*	25.3±1.2	30.2±1.8	26.6±0.4	16.6±1.0	23.7±0.8	26.8±1.1	34.8±4.5	24.3±3.7
Volume (m^3^ ha^-1^)*	279.9±12.1	369.0±23.0	285.6±6.1	144.6±8.5	210.3±3.5	235.3±20.9	388.0±37.9	208.6±29.4

*Mean ± standard deviation, derived from the measurement of five plots with ±500 m^2^ in each planting.

### Data obtaining

The data came from rigorous scaling on a 460 felled tree stems from 388 trees/stumps. Bark diameters (cm) were measured with a bevel gauge, at different tree heights: cutting height (±0.15 m); 0.4; 0.7; 1.0; 1.3; and 2.0 m, counted from ground level. From this point on, measurements were taken every two meters to the minimum commercial diameter with a 5 cm bark. The heights (m) at which the bark diameters of 10 cm, 15 cm, and 20 cm occurred were also obtained. The bark thickness (cm) was measured in a slice of the bark using a bevel gauge in each position of the stem that had the bark diameter measured. The diameter without bark (cm) was obtained by subtracting the bark diameter by multiplying twice the bark thickness.

**Fig 1:**
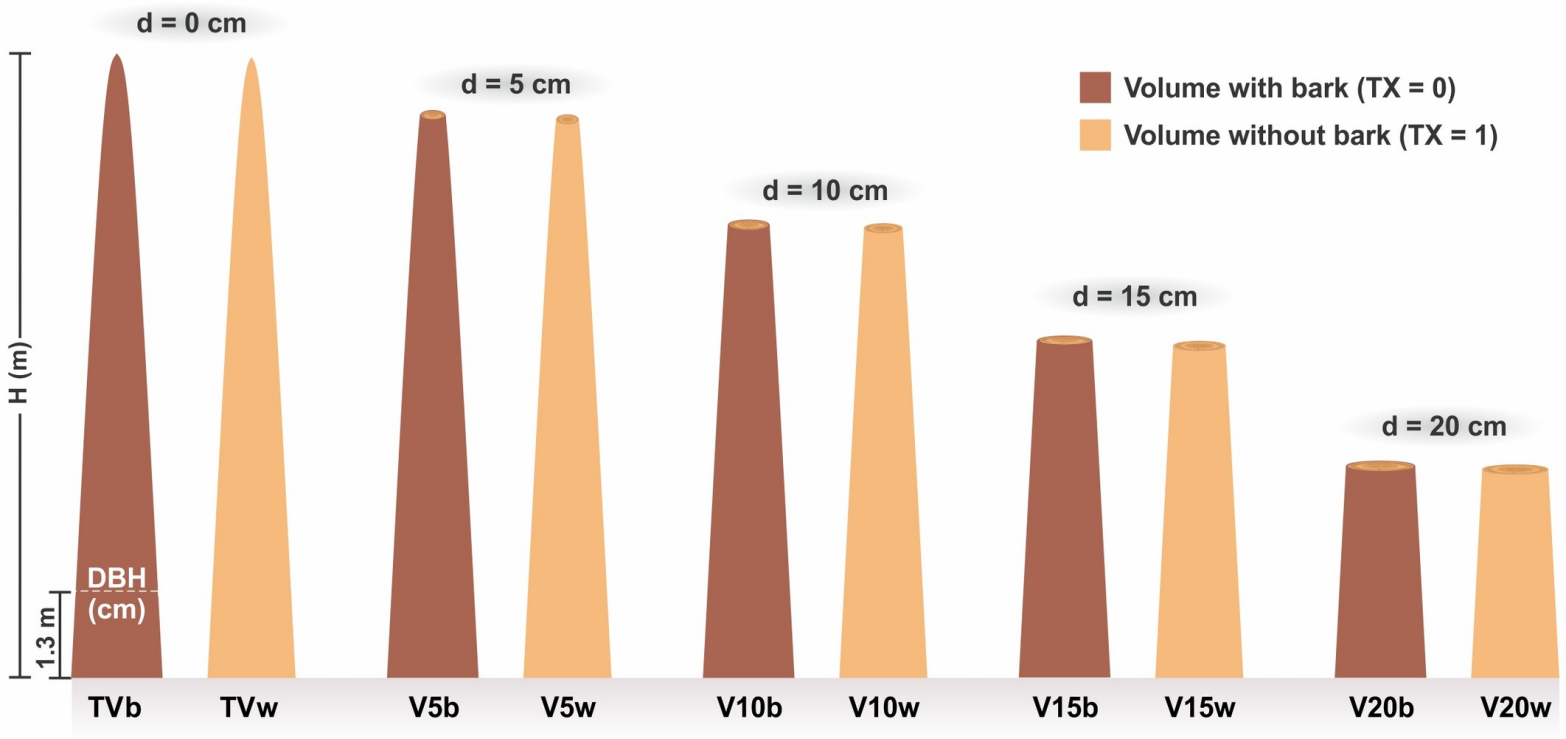
Schematic representation of the multiple possible volumes to be obtained at each cost. H (m) = total height; DBH (cm) = diameter at breast height; d = commercial diameter; TVb and TVw = total volume with and without bark; V5b and V5w = volume with and without bark up to the commercial diameter of 5 cm; V10b and V10w = volume with and without bark up to the commercial diameter of e 10 cm; V15b and V15w = volume with and without bark up to the commercial diameter of 15 cm; V20b and V20w = volume with and without bark up to the commercial diameter of 20 cm.

The volumes between the diameters measured at the stem (sections), with and without bark, were obtained by the Smalian method. The volume of the stem tip was obtained by multiplying half of the sectional area in the 5 cm diameter by the tip length. The total volume of each tree, with and without bark, was obtained by summing the volume of the sections with the volume of the tip. Commercial volumes with and without bark were also obtained from each tree to commercial bark diameters of 5 cm, 10 cm, 15 cm, and 20 cm, provided that the stem had a minimum log length of 1.0 m or more (Fig 1). Thus, up to 10 volumes were obtained for each tree, limited by the diameter of the trees.

### Data analysis

**Fig 2:**
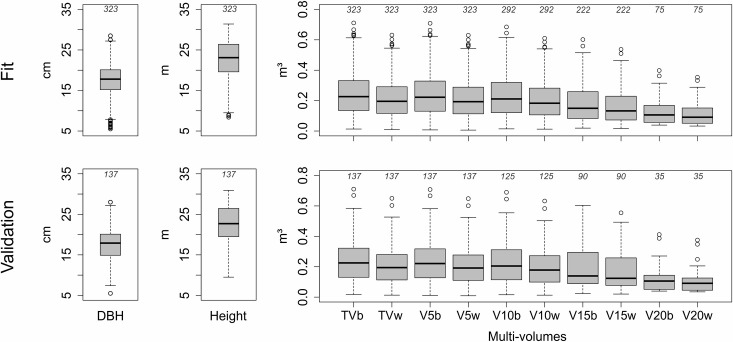
Boxplot of variables used in the fit and validation steps. DBH = diameter at breast height; TVb to V20w = Multi-volumes specified in Fig 1. Values above the bars indicate the number of observations for each variable.

Initially, the 460 tree stems rigorous scaling were randomly divided into two datasets, one intended for fitting regression models and ANN training (70%) and another for validation (30%). The first was composed of the different volumes obtained in 323 tree stems, totalizing 2470 observed volumes. The validation was composed of volumes obtained in 137 tree stems, totaling 1048 observed volumes. Boxplots of the variables used in the fit and validation steps are shown in Fig 2. The data file used in each stage (training and validation) is available as Support Information.

These data were used for the modeling of the multiple volumes of the stem by different strategies: Schumacher and Hall [8] model fitting, separately adjusted for each volume of interest (total and a merchantable volume, with and without bark); multi-volume single model fitting proposed by Leite et al. [20]; and ANNs training to estimate multi-volumes of the stem.

### Schumacher and Hall model

The Schumacher and Hall model [8] was fitted for each of the ten volumes separately, with the support of the R software, package nlme [42]. Among the many existing models for expressing wood volume as a function of diameter and height. The model proposed by Schumacher and Hall [8] is one of the most widespread in the forestry area due to its statistical properties since it almost always results in unbiased estimates [29, 37, 43].

The functional form of the Schumacher and Hall model is given by:

$$V=\beta_{0}.DBH^{\beta_{1}}.H^{\beta_{2}}+\varepsilon$$ (1)

Wherein: V = volume (m^3^); *β*_*i*_ = regression coefficient; DBH = diameter obtained at 1.3 m height from the ground level (cm); H = total height (m); *ε* = random error.

### Multi-volume model

The fitted multi-volume model was proposed by Leite et al. [20], with the support of the R software, package nlme [42]. Whose functional form is:

$$V=\beta_{0}.DBH^{\beta_{1}}.H^{\beta_{2}}.\left[\exp^{\frac{\beta_{3}.TX}{DBH}}\right].\left[1-{\left(\frac{d}{DBH}\right)}^{1+\beta_{4}.d}\right]+\varepsilon$$ (2)

Wherein: *β*_*i*_ = regression coefficients; DBH = diameter at breast height of 1.3 m ground level (cm); H = total height (m); TX = binary variable, which takes values 0 and 1; d = commercial diameter (cm); *ε* = random error. If TX = 0, the equation gives the volume with bark; if TX = 1, it provides the volume without bark. If d = 0, the adjusted equation provides the total volume. If d assumes any other value, the equation provides the volume up to the stipulated diameter.

Table 2 shows how the data were organized to fit the multi-volume model is shown.

**Table 2:** Data used for fitting the multi-volume model.

V	DBH	H	TX	d
TVb_1_	DBH_1_	H_1_	0	0
TVw_1_	DBH_1_	H_1_	1	0
V5b_1_	DBH_1_	H_1_	0	5
V5w_1_	DBH_1_	H_1_	1	5
V10b_1_	DBH_1_	H_1_	0	10
V10w_1_	DBH_1_	H_1_	1	10
V15b_1_	DBH_1_	H_1_	0	15
V15w_1_	DBH_1_	H_1_	1	15
V20b_1_	DBH_1_	H_1_	0	20
V20w_1_	DBH_1_	H_1_	1	20
…	…	…	…	…
V20w_n_	DBH_n_	H_n_	1	20

V = volume (m^3^); DBH = diameter obtained at 1.3 m height from the ground level (cm); H = total height (m); TX = binary variable, which takes values 0 and 1; d = commercial diameter (cm); n = number of tree stem; TVb to V20w = Multi-volumes specified in Fig 1.

### ANNs training

The ANNs training was performed using the Intelligent Problem Solver tool from the Statistica 7.0 software [44]. This tool allows optimizing the ANN architecture by automatically setting the best number of neurons in the hidden layer and the best activation functions of the hidden and output layers, choosing the one with the least possible error, and is widely used by the scientific community [45–47].

For this reason, we used the most popular neural network layout, the Multilayer Perceptron (MLP). The main algorithm for MLP training is backpropagation (BP), which has been described in detail by Goh [48]. BP uses the momentum term to control the ANN learning rate. This momentum causes weight changes to be affected by the size of the previous weight changes that are used to avoid overfitting. The learning rate tells the network how slowly to progress. The weights are updated by a fraction of the calculated error each time to prevent the network from making large swings about the best values without ever getting it right [34]. The training of the MLP network by BP involves three stages [25, 49]: (i) the feedforward of the input training pattern, (ii) the calculation and backpropagation of the associated error, and (iii) the adjustment of the weights.

In this work, 1,000 MLP networks with one hidden layer containing a maximum of ten neurons were trained. The backpropagation training algorithm was used, and the logistic function performed the activations of the hidden and output layers. The input variables were DBH, H, TX, and d (previously defined), with data organized similarly to that adopted for multiple volumes (Table 2). The software retained the five best performing training ANNs.

### Assessing the fit quality of the regression and ANN training models

The quality of the estimates generated by the different strategies was analyzed by the following criteria: coefficient of determination–R^2^ (Eq 3), root mean square error–RMSE(%) (Eq 4), mean absolute error–MAE(%) (Eq 5), mean bias error–MBE(%) (Eq 6), residual (%) (Eq 7), and graphical analysis for observed and estimated values and distribution of residuals [14, 35, 40, 50]. These analyses were performed using the R software [51] and Microsoft Excel.

$$R^{2}=1-\frac{\sum_{i=1}^{n}{\left(y_{i}-{\hat{y}}_{i}\right)}^{2}}{\sum_{i=1}^{n}{\left(y_{i}-\bar{y}\right)}^{2}}$$ (3)

$$RMSE(\%)=\frac{100}{\bar{y}}\sqrt{\frac{\sum_{i=1}^{n}\left(y_{i}-{\hat{y}}_{i}\right)}{n}}$$ (4)

$$MAE(\%)=\frac{100}{\bar{y}}\frac{\sum_{i=1}^{n}\left|y_{i}-{\hat{y}}_{i}\right|}{n}$$ (5)

$$MBE(\%)=\frac{100}{\bar{y}}\frac{\sum_{i=1}^{n}\left(y_{i}-{\hat{y}}_{i}\right)}{n}$$ (6)

$$Residual(\%)=\frac{\left(y_{i}-{\hat{y}}_{i}\right)}{y_{i}}100$$ (7)

Wherein: yi = observed value of the ith variable; ${\hat{y}}_{i}$ = estimated value of the ith variable; $\bar{y}$ = observed mean of the variable; n = sample size.

### Validating equations and ANNs

For validation of the generated equations and trained ANNs, estimates of multi-volumes were generated using the unused dataset for model fit and ANNs training. The quality of the estimates in the validation step was analyzed by the same criteria adopted for assessing the fit and training quality of the ANNs.

Additionally, to confirm the hypotheses tested, the t-test (α = 0.05) was performed between the volume obtained in the rigorous scaling and the estimated volume by each strategy with the validation data. In the case of ANNs, the t-test was performed for the best performing ANN in the validation step. This analysis was performed on Rbio software [52].

## Results

### Schumacher and Hall equations

Performance on volume estimates was variable by fitting the Schumacher and Hall volumetric model for each volume separately (Table 3, S1 and S2 Figs). The best estimates were obtained for total and commercial volume up to 10 cm diameter, which presented the best statistics, both in the model fitting and validation. Overall, the best quality of estimates was proportional to the decrease in the minimum commercial diameter.

**Table 3:** Statistics obtained in the fit and validation of the Schumacher and Hall model to estimate multi-volumes of eucalyptus trees.

V	*β*_*0*_	*β*_*1*_	*β*_*2*_	Fit				Validation			
				R^2^	RSME	MAD	MBE	R^2^	RSME	MAD	MBE
					(%)	(%)	(%)		(%)	(%)	(%)
TVb	6.85x10^-5^	1.4120	1.3260	0.970	10.66	7.29	-0.54	0.971	10.34	6.89	-0.15
TVw	4.64x10^-5^	1.4161	1.4014	0.969	11.10	7.72	-0.54	0.971	10.68	7.18	-0.18
V5b	5.83x10^-5^	1.4330	1.3520	0.969	11.14	7.78	-0.72	0.970	10.77	7.36	-0.02
V5w	4.01x10^-5^	1.4366	1.4245	0.968	11.53	8.18	-0.71	0.970	11.04	7.56	-0.28
V10b	2.20x10^-5^	1.6520	1.4200	0.947	14.41	10.71	-1.19	0.949	14.62	10.78	-1.09
V10w	1.56x10^-5^	1.6480	1.4910	0.946	14.66	10.96	-1.18	0.951	14.66	10.80	-1.07
V15b	9.01x10^-7^	2.4120	1.5770	0.873	25.38	20.17	-2.15	0.869	25.33	19.68	0.14
V15w	6.43x10^-7^	2.4130	1.6420	0.873	25.76	20.51	-2.16	0.874	25.38	19.68	0.21
V20b	1.31x10^-8^	4.1830	0.9340	0.747	34.34	27.81	-1.34	0.768	38.56	27.85	-1.96
V20w	7.62x10^-9^	4.2220	1.0230	0.746	35.02	28.37	-1.34	0.774	38.89	27.81	-1.76

Wherein: V = Volume; TVb to V20w = Multi-volumes specified in Fig 1; *β*_*0*_, *β*_*1*,_ *β*_*2*_ = model coefficients; R^2^ = coefficient of determination; RMSE(%) = root mean square error; MAE(%) = mean absolut error; MBE(%) = mean bias error.

**Fig 3:**
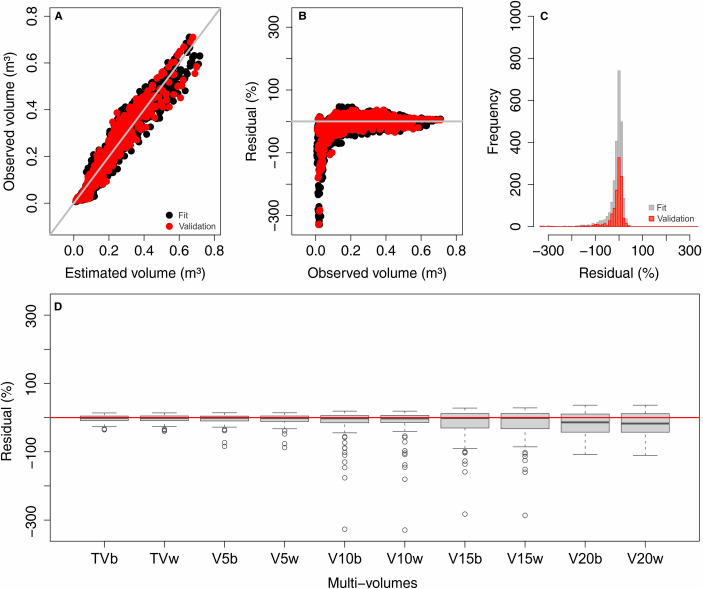
(A) Correlation between observed and estimated volume, (B) residual distribution, and (C) residual histogram for the fit and validation steps from the Schumacher and Hall equations. (D) Boxplot of residues for multi-volumes separately at the validation step. TVb to V20w = Multi-volumes specified in Fig 1.

The Schumacher and Hall equations demonstrated good performance in the graphical analysis of the multi-volumes jointly assessed in the fit and validation steps. The estimates showed a good distribution of residuals and were not biased, except for volumes smaller than 0.15 m^3^, which were overestimated (Fig 3A–3C). The recalculated fit and validation statistics for the multi-volumes jointly were adequate, with R^2^ = 0.947, RMSE(%) = 15.29, MAE(%) = 11.10 and MBE(%) = -1.26 for fit, and R^2^ = 0.948, RMSE(%) = 15.17, MAE(%) = 11.70, and MBE(%) = -0.02 for validation. These figures indicate that the strategy used was efficient in estimating multi-volumes, and this is supported by the higher distribution of residuals around zero for each multi-volume in the validation (Fig 3D). The distribution pattern is more influenced by the commercial diameters adopted than by the presence or not of bark in the stem. The volumes for the commercial diameters of 15 and 20 cm showed higher dispersion and a slight tendency to overestimate the values in the validation.

### Multi-volume equation

Eq 8, from the multi-volume model fitting (Eq 2), performed well for the estimation of the total and commercial volumes (up to different minimum diameters), with and without bark (R^2^ = 0.932; RMSE(%) = 17.26; MAE(%) = 12.28; MBE(%) = 0.41). When applying the equation to validation data, a behavior similar to that observed in the fit step was verified (R^2^ = 0.938; RMSE(%) = 16.56; MAE(%) = 11.83; MBE(%) = 1.81), indicating that the equation is adequate to estimate the volume of parts of the eucalyptus stem through a single equation.

$$V=0.0001486.DBH^{1.0970}.H^{1.3919}.\left[{exp}^{\frac{-2.5488.TX}{DBH}}\right].\left[{1-(\frac{d}{DAP})}^{1+0.1529.d}\right]$$ (8)

**Fig 4:**
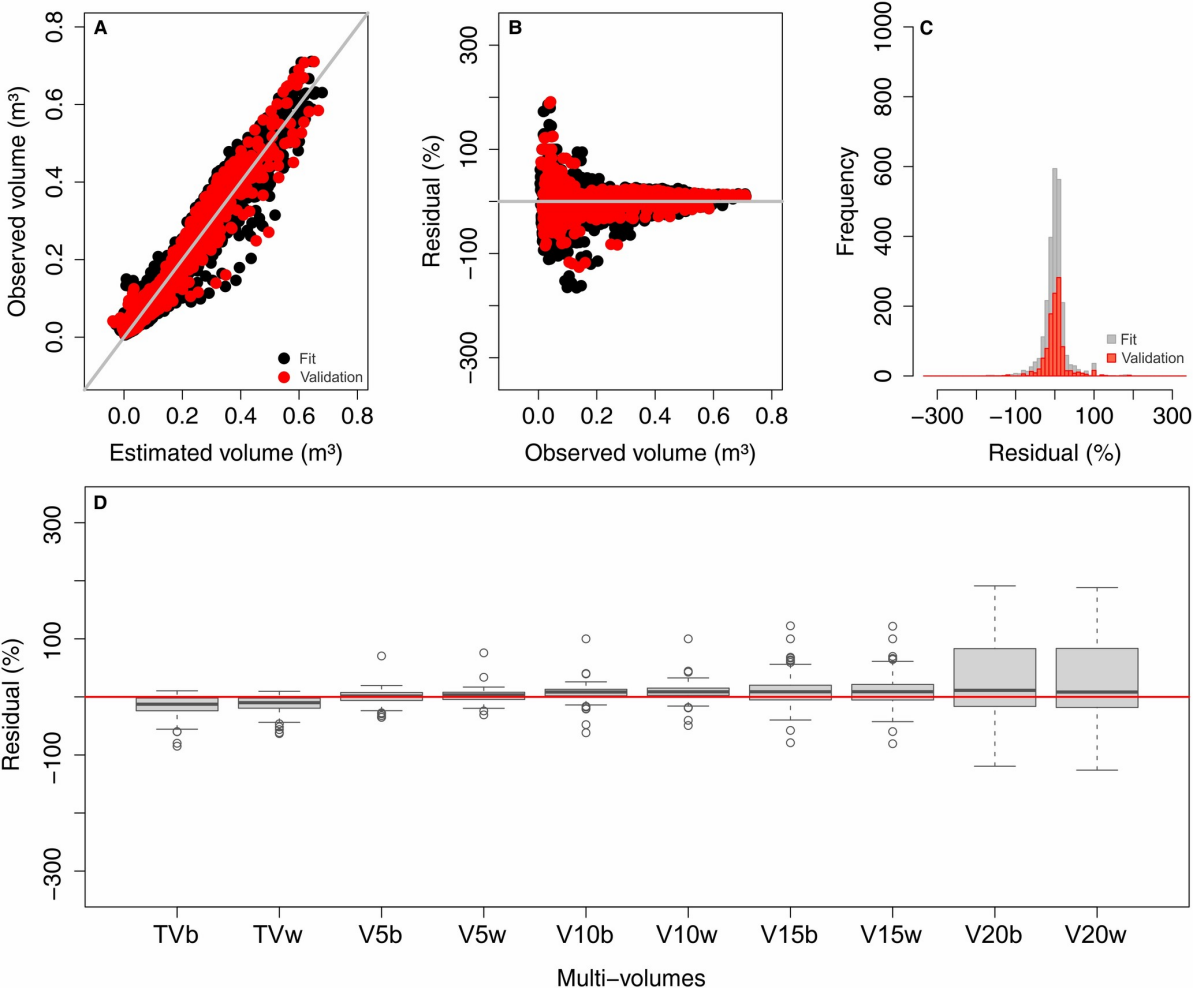
(A) Correlation between observed and estimated volume, (B) residual distribution, and (C) residues histogram for the fit and validation steps from the Multi-volume equation. (D) Boxplot of residuals for multi-volumes separately at the validation step. TVb to V20w = Multi-volumes specified in Fig 1.

Overall, the equation provided good estimates in the fit and validation steps without bias in the estimates (Fig 4A–4C). At validation, the boxplot of the residuals for multi-volumes separately shows a higher distribution of residuals around zero (Fig 4D), which indicates that this strategy was also efficient. As for the Schumacher and Hall equations, the pattern of this distribution is more influenced by different commercial diameters adopted than by the presence or not of bark in the stem. A slight bias in overestimating total volumes and a higher bias in underestimating volumes for the 20 cm commercial diameter were observed. This fact revealed a higher dispersion between the observed and estimated values.

### ANNs

ANNs have also been shown to be an efficient strategy for estimating multi-volumes of eucalyptus trees. The best performing ANNs had variable architecture in the number of neurons in the input layer (3–4) and the intermediate layer (4–7) (Table 4). The weight of the input variable followed the order: DBH > d > H > TX, where the TX variable does not influence the volume estimates in ANN 1. The exclusion of this variable from the input layer impaired the quality of the estimates generated by this network (S3, S4, S5 and S6 Figs), causing an increased in RSME (%) and MAD (%). Overall, all ANNs showed superior performance in the validation.

**Table 4:** Characteristics of the best performing artificial neural networks (ANNs) and statistics obtained in the training and validation to estimate multi-volumes of eucalyptus trees.

ANN	Arch	Input variables*	Training				Validation			
			R^2^	RSME	MAD	MBE	R^2^	RSME	MAD	MBE
				(%)	(%)	(%)		(%)	(%)	(%)
1	3-5-1	DBH, d, H	0.946	15.33	11.09	0.01	0.946	15.35	10.93	0.46
2	4-4-1	DBH, d, H, TX	0.957	13.76	9.64	-0.11	0.957	13.68	9.22	0.38
3	4-5-1	DBH, d, H, TX	0.958	13.47	9.41	-0.25	0.960	13.24	8.91	0.41
4	4-6-1	DBH, d, H, TX	0.958	13.49	9.48	-0.20	0.959	13.40	8.96	0.38
5	4-7-1	DBH, d, H, TX	0.958	13.52	9.54	-0.05	0.958	13.51	9.17	0.33

Arch = ANN architecture; DBH = diameter at breast height (m); d = commercial diameter (cm); H = total height (m); TX = binary variable that takes values 0 and 1

* = ranked according to the weight of the variables in each ANN; R^2^ = coefficient of determination; RMSE(%) = root mean square error; MAE(%) = mean absolut error; MBE(%) = mean bias error.

**Fig 5:**
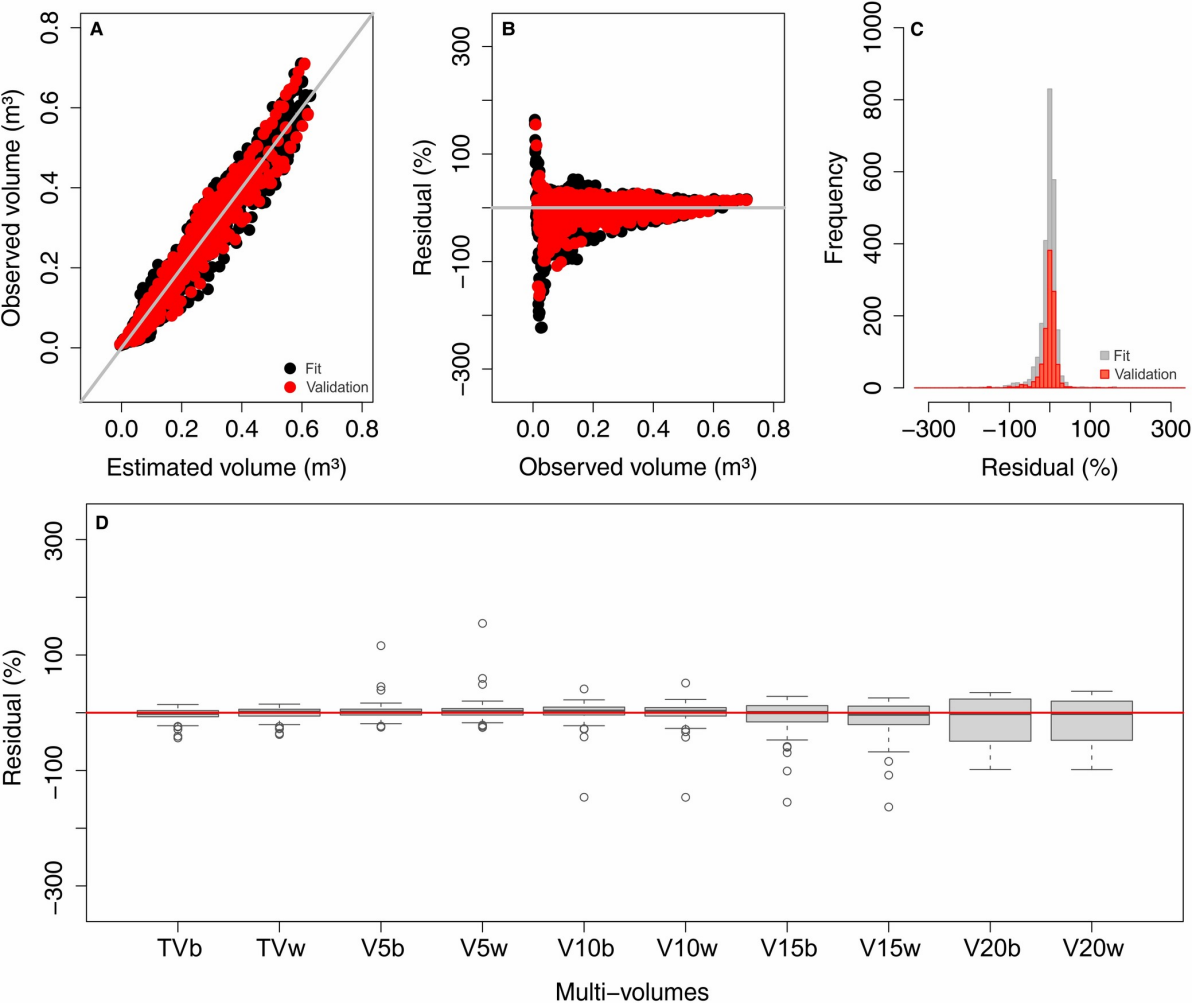
(A) Correlation between observed and estimated volume, (B) residual distribution, and (C) residual histogram for fit and validation steps from the best ANN (ANN 3). (D) Boxplot of residues for multi-volumes separately in the validation step. TVb to V20w = Multi-volumes specified in Fig 1.

The ANN 3 presented statistics slightly better than the others. This network provided unbiased estimates (Fig 5A–5C) and less dispersion around zero for all volumes when compared with the estimates obtained by Schumacher and Hall and multi-volume equations (Figs 3 and 4), supporting the statistics obtained. In the validation, despite the ANN 3 superiority, the volume estimates for 20 cm diameters were less accurate than the volume up to the other assessed diameters, with a higher dispersion between the observed and estimated values (Fig 5D). Regarding the other strategies, ANN 3 provided better graphic patterns.

### Efficiency of strategies in estimating multi-volumes

**Fig 6:**
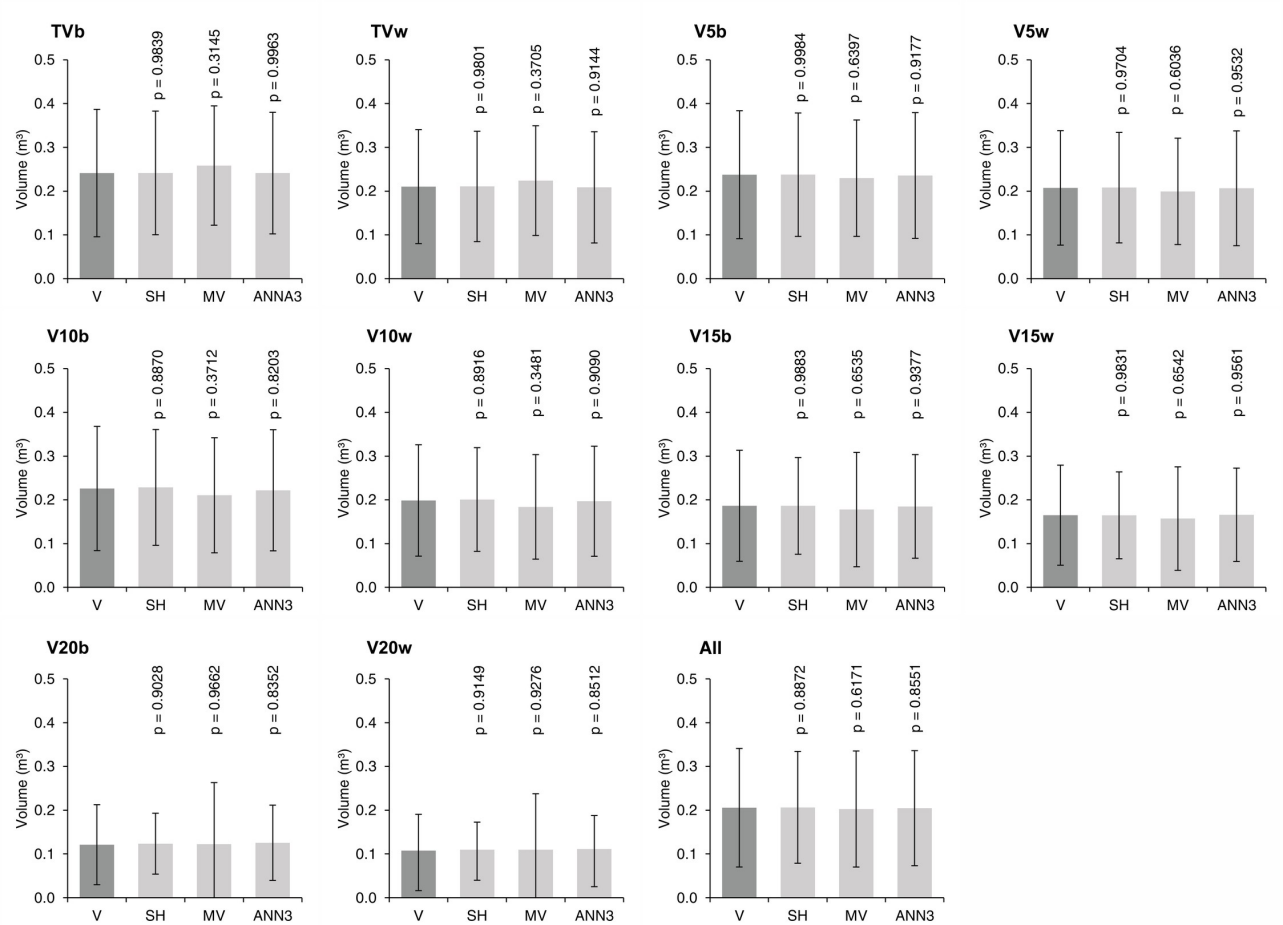
**The t-test for multi-volume means estimated by Schumacher and Hall equations (SH), multi-volume equation (MV), and artificial neural network (ANN3) (light gray bars) in relation to the actual volume obtained in rigorous scaling (V) (dark gray bars) at the validation step.** Error bars indicate the standard deviation. p = indicates the probability by the t-test of the respective volume estimated with the actual volume. TVb to V20w = Multi-volume specified in Fig 1.

Test t demonstrated that there were no significant differences between the actual volumes obtained in rigorous scaling and those estimated by the different strategies adopted in the validation (Fig 6). Overall, the standard deviation for the estimated values is similar to those observed for the actual volumes. This finding reveals that the estimated volumes are consistent with the actual volumes.

## Discussion

These findings demonstrate that the strategies used for modeling different tree stem volumes (Schumacher and Hall volume-specific models, multi-volume model, and ANNs) were efficient. No significant differences were detected between the estimated volumes and the actual volume (Fig 6), and all strategies showed high accuracy and adequate graphic patterns. However, for larger commercial diameters, the estimates proved to be less accurate, with higher dispersion between observed and estimated values. One possible reason for this result is the higher irregularity in the form of the trunk base [6, 20, 53], which increases the natural volume variability in this portion of the stem and may weaken the relationship between the independent variable and the dependent variables. This fact impairs the predictive ability of the models. Therefore, when estimating these volumes, one should pay close attention to possible inconsistencies in the estimates, regardless of the modeling strategies evaluated.

Each of the strategies for estimating volumes has advantages and disadvantages in some situations. Adjusting a Schumacher and Hall volume-specific equation for each volume of interest separately improved the evaluated statistics and graphical standards when compared with the single multi-volume model proposed by Leite et al. [20]. However, this strategy would require the use of several equations (ten in this study), which can make it challenging to plan a forest enterprise, due to the cost and time spent adjusting the equations separately [16]. Thus, the forester should consider the benefit that would have more weight in forest planning, either greater accuracy or greater practicality.

In this sense, the ANNs proved to be useful for estimating the multi-volumes of eucalyptus trees simultaneously. The better performance verified for the ANN 3 in the validation (Table 4 and Fig 5) can be explained by the fact that they have simpler topologies between networks, with smaller numbers of neurons in the hidden layer. A reduced number of neurons in the hidden layer avoids the overfitting problem, which consists of over-learning the information contained in the data offered to ANNs [28]. ANNs can suffer from over-fitting, but the selection of suitable architecture may avoid this problem by using training and testing data sets [33]. Simpler topologies also facilitate the search and optimization of the configuration for a given task.

ANNs stood out for their higher accuracy in estimates when compared with the Schumacher and Hall volume-specific and multi-volume equations, although no statistical differences on average were detected. Regarding the setting of a Schumacher and Hall volume-specific model for each desired volume, the ANNs reduced the time required to obtain the estimates and the number of equations required. They also provided similar or superior accuracy to that found for the volumetric equations. In practice, accuracy, convenience, and cost-effectiveness play a crucial role when deciding which methods to utilize for forest inventory [33].

The ANNs are the best alternative to be used by companies or forestry enterprises due to their high potential to replace traditional volumetric models. This method maintains the precision in volume estimates and is convenient and efficient in obtaining results. It can help reduce inventory costs and time to make estimates available [30, 54, 55]. These results were already expected due to the several advantages shown by the ANNs, such as their massive and parallel-distributed structure (layers), the ability to learn and generalize, which enables them to solve complex problems and the fault and noise tolerance. In addition, no need to assume an underlying data distribution, as is usually done in statistical modeling; the possibility of modeling several variables and their non-linear relationships; the possibility of modeling using categorical variables, besides quantitative variables; and neurobiological analogy [33–35, 55].

Therefore, among the multi-volume modeling strategies evaluated here, the use of ANNs is the most promising. Reliable and accurate volume estimating is essential for the forestry company to make a correct assessment of the wood stock and to analyze the productive potential of a forest for multiple uses [4, 7, 56]. The optimization of the techniques used to obtain volumetric estimates has been increasingly frequent in sustainable forest management. Thus, the development of methods to estimate multi-volumes along the tree stem is necessary by combining precise and practical strategies.

However, the application of ANNs to practical forestry is still immature [33]. Using artificial intelligence (AI) techniques demands much training time and can easily incur data overfitting. Furthermore, the most critical decision support systems in forestry are not yet able to handle AI [35, 57]. The adoption of multi-volume ANNs in wood inventories and management plans in other regions of Brazil for other species and silvicultural conditions is made possible by the continuous training of new ANNs as new data are obtained. Depending on the nature of the data (broader scale), a single ANN may be used with efficiency and accuracy for different regions. Therefore, multi-volume ANNs should be the subject of future research assessing other experimental conditions and species/clones, different ANN architectures, or including other quantitative variables and categorical (qualitative) variables.

## Conclusion

The multi-volume model had the most considerable advantage in volume estimation practicality, while the volume-specific models were more efficient in the accuracy of estimates.

Under the conditions tested here, the ANNs are more suitable than regression models in the estimation of multi-volumes of eucalyptus trees, revealing higher accuracy and practicality.

## Supporting information

S1 Fig Correlation between observed and estimated volume, residual distribution, and residual histogram for the fit and validation steps from the Schumacher and Hall equations. Boxplot of residues for multi-volumes separately at the validation step. TVb to V10w = Multi-volumes specified in Fig 1.
(JPG)

S2 Fig Correlation between observed and estimated volume, residual distribution, and residual histogram for the fit and validation steps from the Schumacher and Hall equations. Boxplot of residues for multi-volumes separately at the validation step. V15b to V20w = Multi-volumes specified in Fig 1.
(JPG)

S3 Fig Correlation between observed and estimated volume, residual distribution, residual histogram for fit and validation steps and boxplot of residues for multi-volumes separately in the validation step from the ANN1. TVb to V20w = Multi-volumes specified in Fig 1.
(JPG)

S4 Fig Correlation between observed and estimated volume, residual distribution, residual histogram for fit and validation steps and boxplot of residues for multi-volumes separately in the validation step from the ANN2. TVb to V20w = Multi-volumes specified in Fig 1.
(JPG)

S5 Fig Correlation between observed and estimated volume, residual distribution, residual histogram for fit and validation steps and boxplot of residues for multi-volumes separately in the validation step from the ANN4. TVb to V20w = Multi-volumes specified in Fig 1.
(JPG)

S6 Fig Correlation between observed and estimated volume, residual distribution, residual histogram for fit and validation steps and boxplot of residues for multi-volumes separately in the validation step from the ANN5. TVb to V20w = Multi-volumes specified in Fig 1.
(JPG)

S1 Table Data file used in training stage for fit of models used in this manuscript. (XLSX)

S2 Table Data file used in validation stage for fit of models used in this manuscript. (XLSX)
