# Supplementary figures and images for: Multi-volume modeling of Eucalyptus trees using regression and artificial neural networks
Source: PLoS One. 2020 Sep 11;15(9):e0238703. doi: 10.1371/journal.pone.0238703 (PMC7485850; doi:10.1371/journal.pone.0238703)

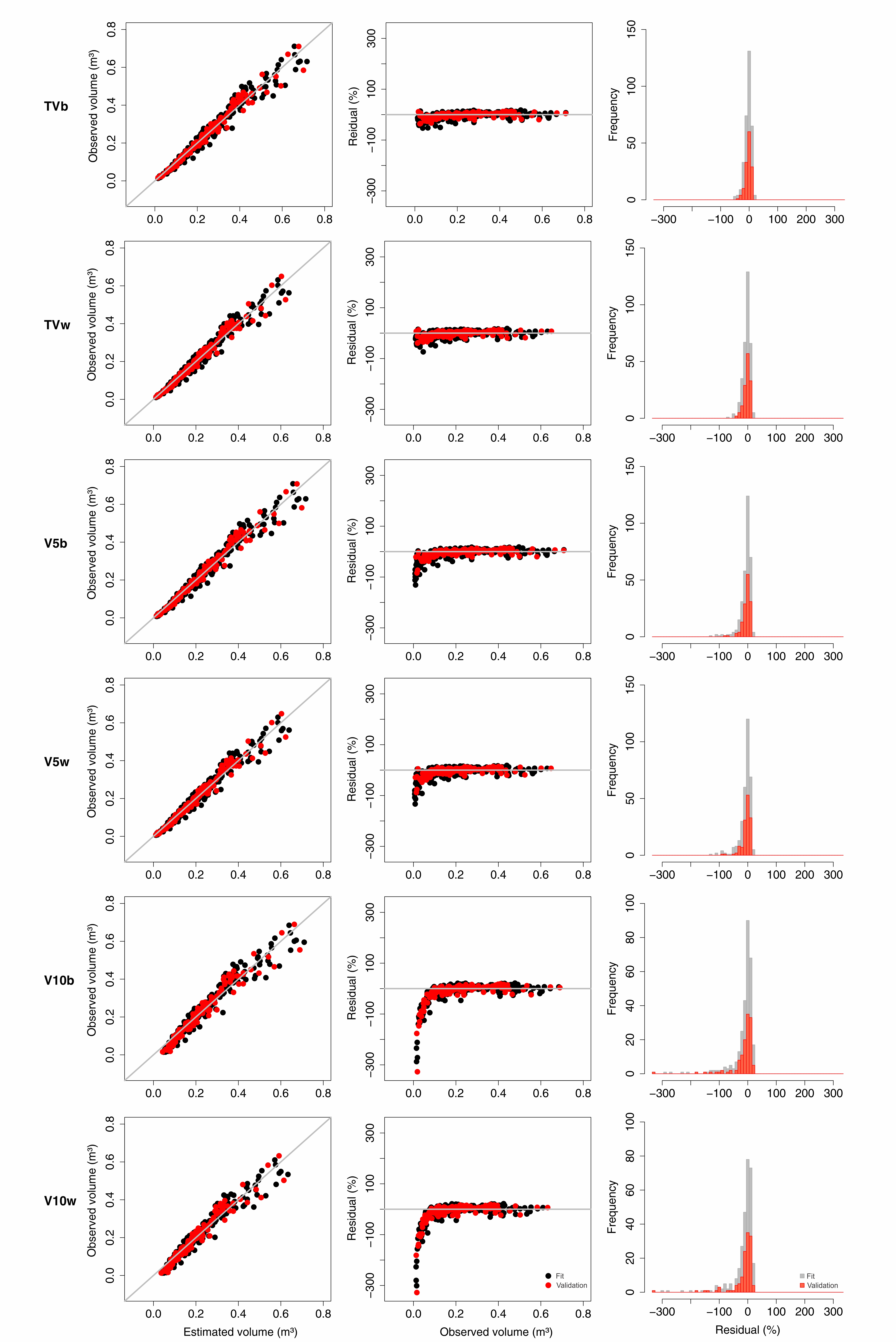

Supplement: S1 Fig — Boxplot of residues for multi-volumes separately at the validation step. TVb to V10w = Multi-volumes specified in Fig 1. (JPG) [file pone.0238703.s001.jpg]

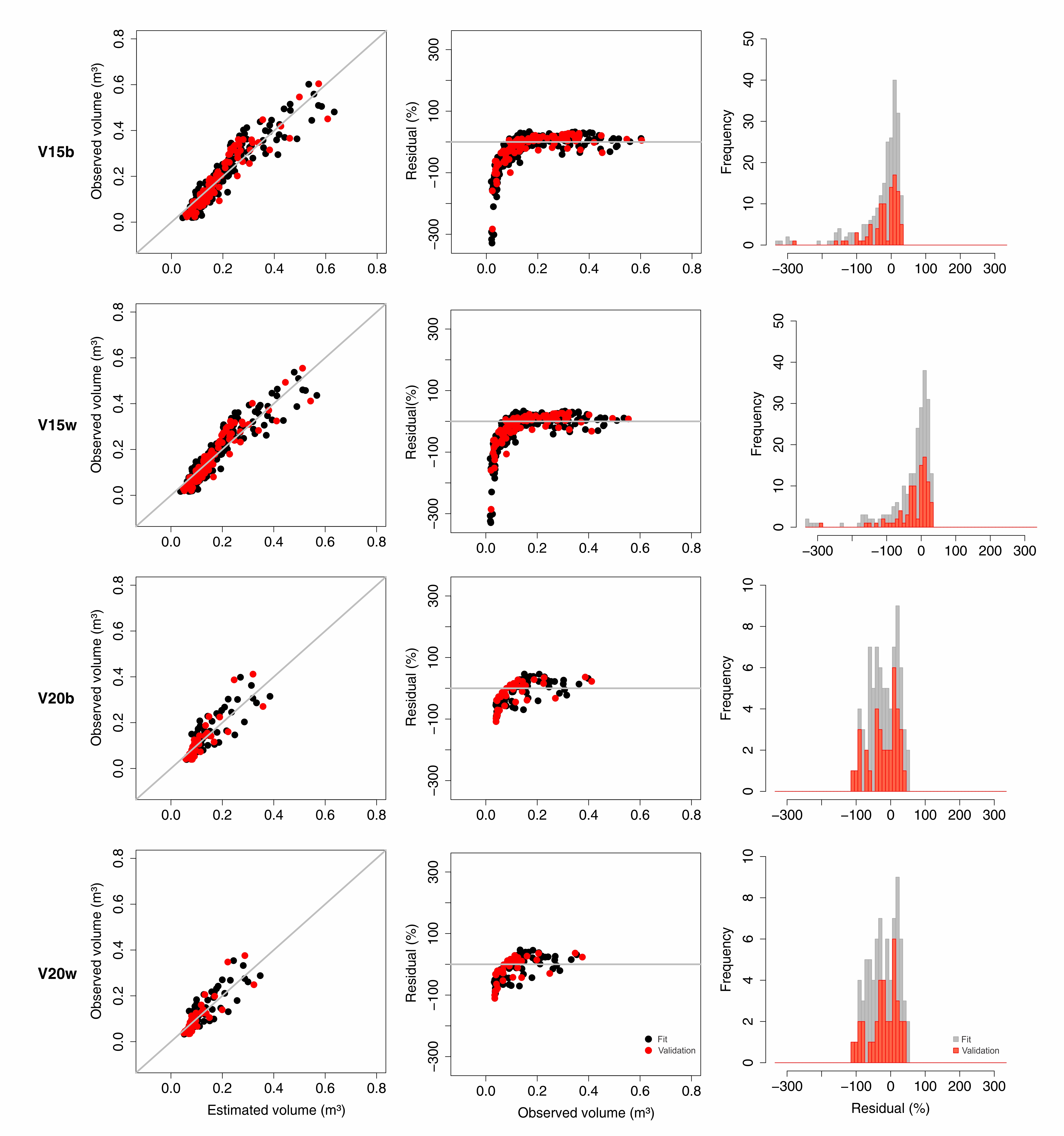

Supplement: S2 Fig — Boxplot of residues for multi-volumes separately at the validation step. V15b to V20w = Multi-volumes specified in Fig 1. (JPG) [file pone.0238703.s002.jpg]

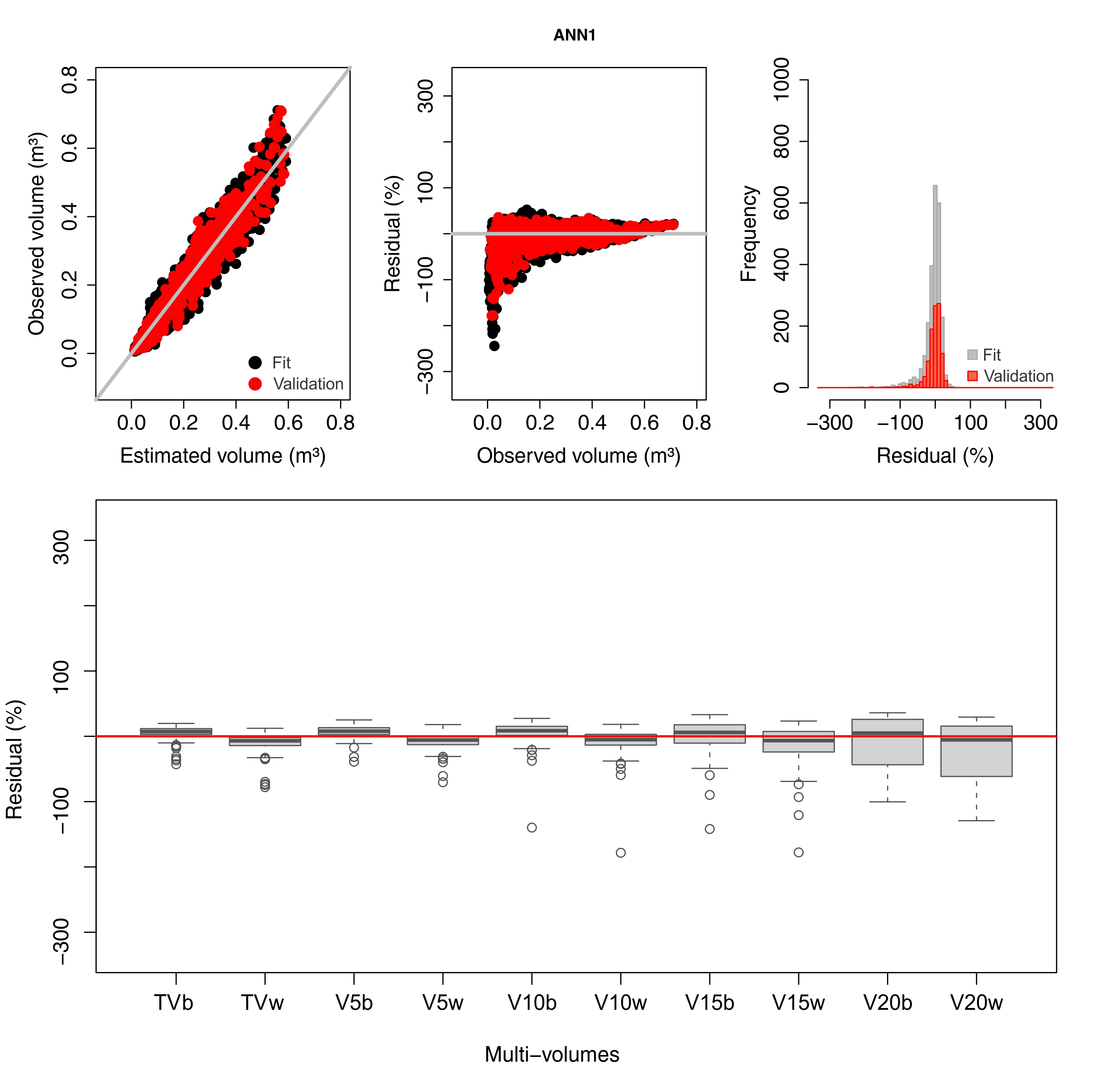

Supplement: S3 Fig — TVb to V20w = Multi-volumes specified in Fig 1. (JPG) [file pone.0238703.s003.jpg]

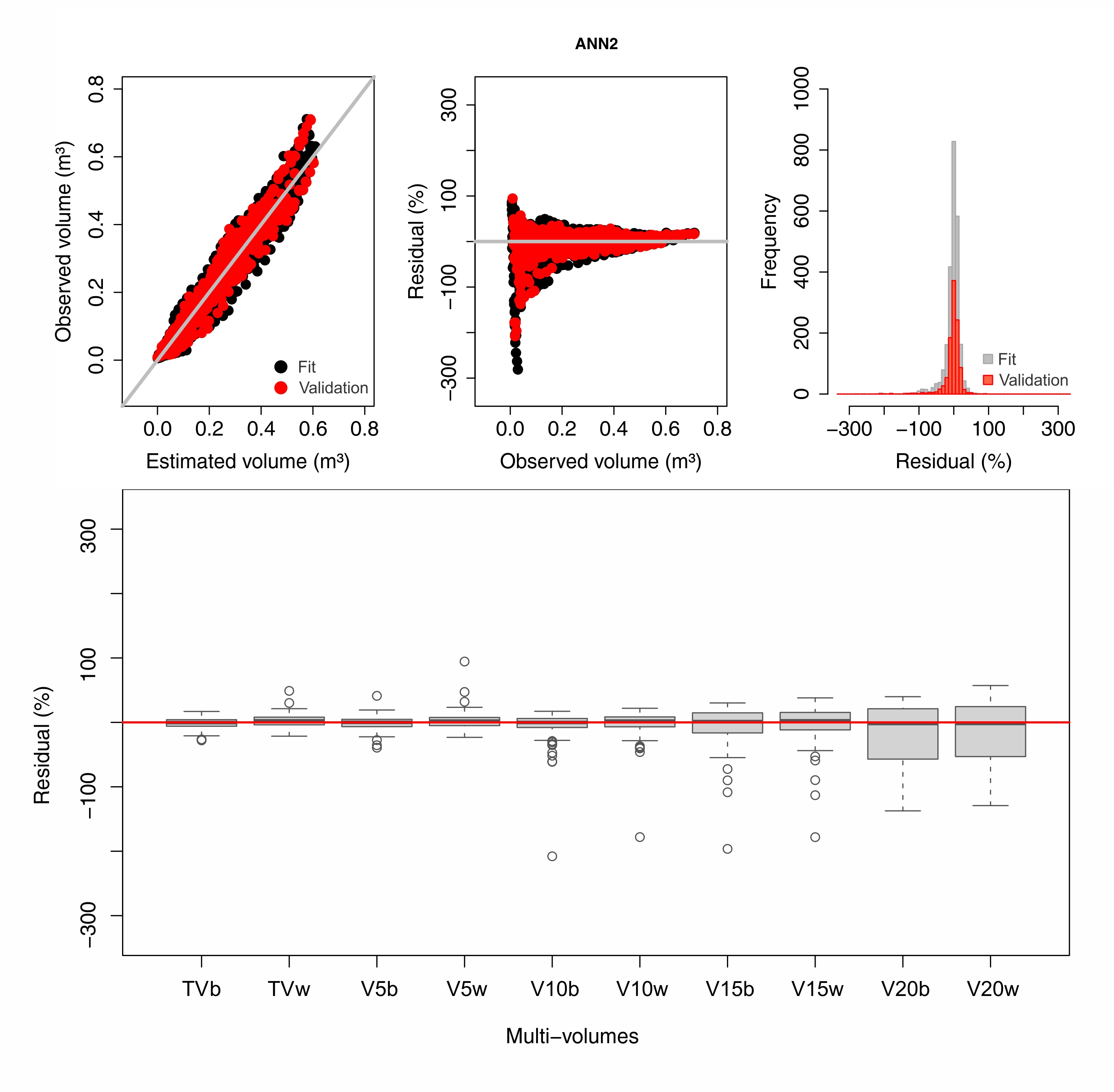

Supplement: S4 Fig — TVb to V20w = Multi-volumes specified in Fig 1. (JPG) [file pone.0238703.s004.jpg]

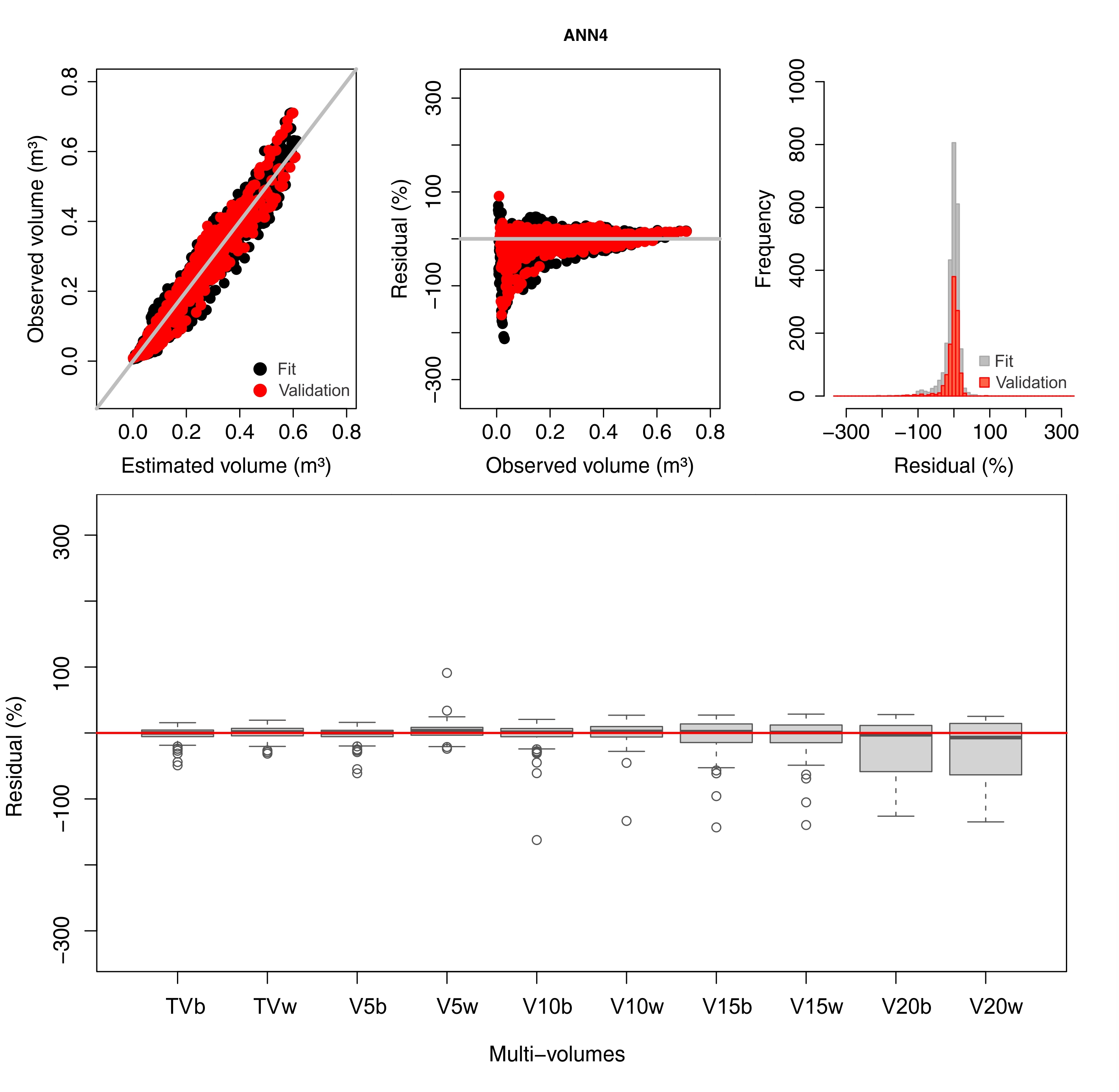

Supplement: S5 Fig — TVb to V20w = Multi-volumes specified in Fig 1. (JPG) [file pone.0238703.s005.jpg]

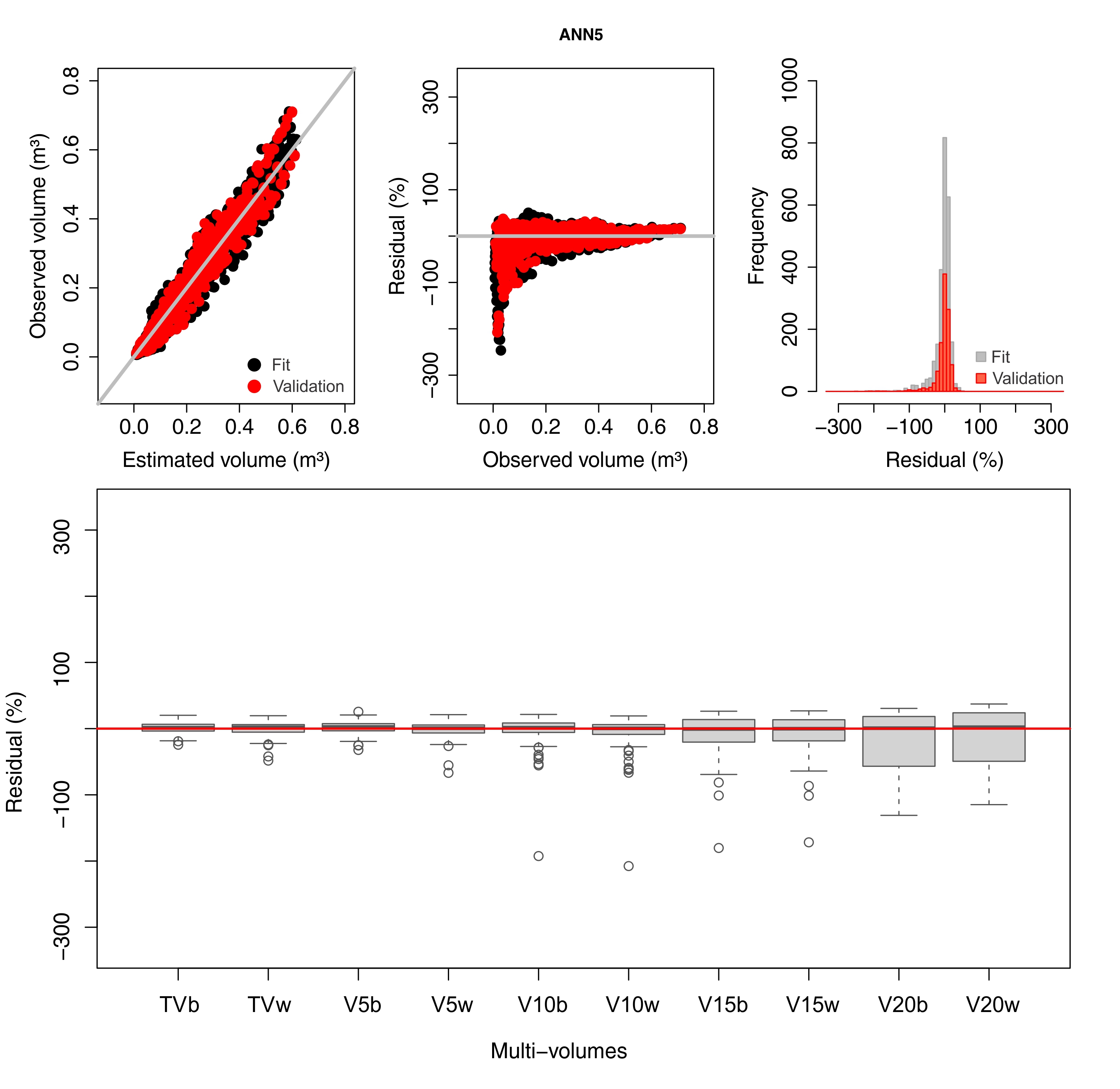

Supplement: S6 Fig — TVb to V20w = Multi-volumes specified in Fig 1. (JPG) [file pone.0238703.s006.jpg]
